# Entanglement revive and information flow within the decoherent environment

**DOI:** 10.1038/srep30710

**Published:** 2016-08-10

**Authors:** Jia-dong Shi, Dong Wang, Liu Ye

**Affiliations:** 1 School of Physics & Material Science, Anhui University, Hefei, 230601, China

## Abstract

In this paper, the dynamics of entanglement is investigated in the presence of a noisy environment. We reveal its revival behavior and probe the mechanisms of this behavior via an information-theoretic approach. By analyzing the correlation distribution and the information flow within the composite system including the qubit subsystem and a noisy environment, it has been found that the subsystem-environment coupling can induce the quasi-periodic entanglement revival. Furthermore, the dynamical relationship among tripartite correlations, bipartite entanglement and local state information is explored, which provides a new insight into the non-Markovian mechanisms during the evolution.

As it is well known, the existence of quantum correlations in composite systems is recognized as one of the most fundamental features of the quantum theory, which can distinguish the quantum realm from the classical one^1^,^2^,^3^,^4^. Conceptually, entanglement^5^, as a special kind of quantum correlation, can be quantified by the Wootters’ concurrence^6^. It is capable of fulfilling a series of realistic tasks of quantum information processing, where it acts as an important physical resource^7^,^8^,^9^,^10^,^11^. However, real quantum systems suffer from inevitable interactions with their surrounding environments. These undesired interactions will lead to decoherence that exponentially damages the quantum correlation^12^,^13^,^14^,^15^,^16^,^17^,^18^,^19^. Hence, it is fundamentally important to understand the dynamics of correlations for a practical open quantum system, and it is also necessary to explore the mechanisms of interactions between the quantum system and environment.

On the other hand, it has been verified that the entanglement in an open quantum system is characterized by some specific phenomena, such as sudden death and revival within the noisy environments^20^,^21^,^22^,^23^,^24^,^25^. Mazzola *et al*. have found that the revivals of entanglement after a finite time period of complete disappearance can be expected when the system is coupled with a common non-Markovian environment. Up to now, a few interpretations of these revivals have been offered: one of the interpretations is that quantum memory of the environment can inherently influence on the coherence of quantum system^26^,^27^; another is that the environment is considered as a control device, and the interactions between quantum system and non-Markovian environment can create quantum correlation^28^,^29^,^30^,^31^,^32^,^33^. Here, we will focus on pursuing the intrinsic mechanisms concerning the revivals of entanglement.

In this paper, we will explore the non-Markovian mechanisms of the revivals of environment by means of the information-theoretic point of view. It turns out that the dynamical interplay between quantum subsystem and noisy environment can induce the quasi-periodic entanglement revivals and information flows. Furthermore, we observe explicit dynamical relationships among the genuine tripartite correlations, bipartite entanglement and local state information. At last, we provide the origin of these relationships in terms of the flow of information among the different constituents involved in the composite system.

## Results

### The model

**Figure 1:**
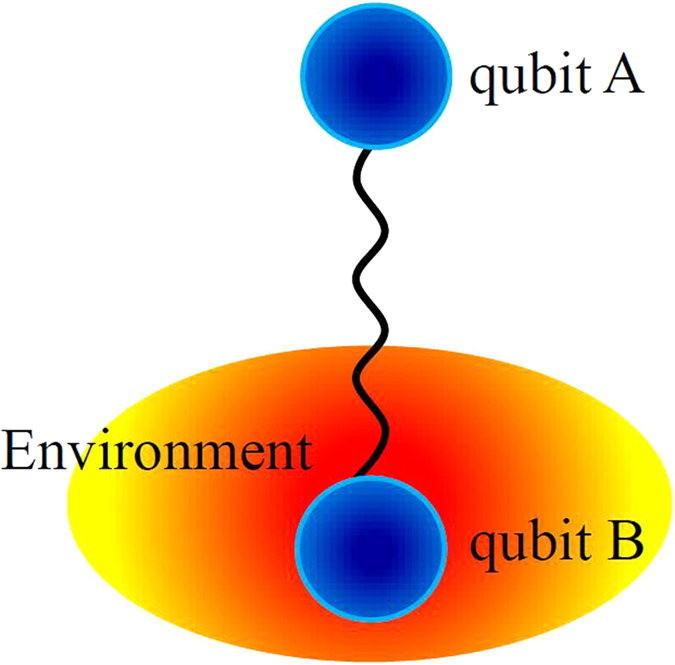
The model sketch of the total system. The two qubits are initially entangled and a local non-Markovian environment only interacts with the qubit *B*, whereas qubit *A* is isolated.

We consider a bipartite system composed of two entangled qubits, one of which (*A*) is isolated while the other (*B*) locally interacts with a noisy environment (*E*). The two qubits are initially prepared in a Bell-like state provided in the next section, while the environment is in the vacuum state. For clarity, the model sketch of the total system is depicted in Fig. 1, where the noisy environment is turned to a non-Markovian environment which can be realized by an electromagnetic field with a single-mode cavity. In fact, the model can be viewed as a tripartite composite system, one part composed by the two qubits and the other part by the non-Markovian environment, which can be similarly treated as a qubit.

In this case, we consider the dynamical map for the single qubit coupled with the local non-Markovian environment, which can be described by the interaction Hamiltonian^23^


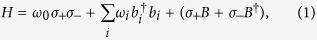


where 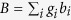 with *g*_*i*_ being the coupling constant, *σ*_±_ are the raising and lowering operators and *ω*_0_ denotes the transition frequency of the two-level qubit. Moreover, the index *i* labels the field modes of the environment with frequency *ω*_*i*_ and 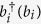 is the modes’ creation (annihilation) operator.

Interestingly, the Hamiltonian in Eq. (1) may describe various systems (see ref. 23) as, for example, a qubit formed by an exciton in a potential that represents a quantum well. Herein, we shall consider the system as a qubit, formed by a two-level atom, and a non-Markovian environment interacting with the qubit. In ref. 34, the local non-Markovian environment at zero temperature can be quantized as an electromagnetic field in a high-*Q* cavity with the following effective spectral density


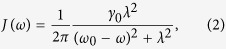


where the parameters *λ* and *γ*_0_ define the spectral width of the environment and the decay rate of the excited state of the qubit, respectively, linked with the environment correlation time *τ*_*B*_, the relaxation time *τ*_*R*_ by *τ*_*B*_≈1/*λ* and *τ*_*R*_≈1/*γ*_*0*_. We provide the detailed proofs of this quantization in the part of Methods. Particularly, their relative magnitudes typically determine a Markovian (*λ* > 2*γ*_0_) and a non-Markovian (*λ* < 2*γ*_0_) regime, respectively^35^. We will focus on the non-Markovian regime in terms of *λ* = 0.1 and *γ*_0_ = 1 in the remainder of our work.

In the following, we use the general notation 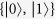 as the computational basis of the qubit. Then, the dynamical map for the single qubit evolved within the non-Markovian regime can be described by the reduced density matrix^23^,^36^


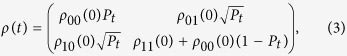


*P*_*t*_ is an oscillation term describing the fact that the decay of the qubit’ excited state is induced by the interaction between the qubit subsystem and the environment. For the effective spectral density *J*(*ω*) in Eq. (2), within the non-Markovian regime *P*_*t*_ can be given by


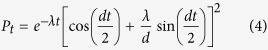


with 
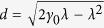
. It is shown that in the non-Markovian regime *P*_*t*_ starts to oscillate, which will lead to a nonmonotonic behavior of the system state.

### Entanglement revival within the decoherent environment

Upon the above preliminaries, we now proceed by discussing about the revivals of entanglement within the local non-Markovian environment. In this regard, the initial state is prepared in the Bell-like state


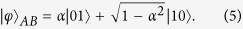


The state is maximally entangled when the condition 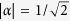 is satisfied.

When only qubit *B* interacts with the local non-Markovian environment *E*, we can obtain the time-evolved state of the composite system in terms of the reduced density matrix for the single qubit shown in Eq. (3) as


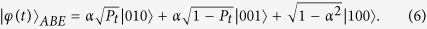


By taking the partial trace of 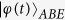 over the degrees of freedom of the environment *E*, one can readily obtain the reduced density matrix *ρ*_*AB*_(*t*) for the qubit subsystem. Subsequently, we will concentrate on observing the dynamics of its entanglement. In this work, the Wootters’ concurrence 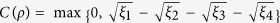 is employed as the measurement of the entanglement^6^. The parameters *ξ*_*i*_ are the decreasing eigenvalues of matrix 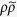, where 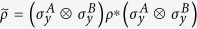 is the spin-flip matrix.

**Figure 2:**
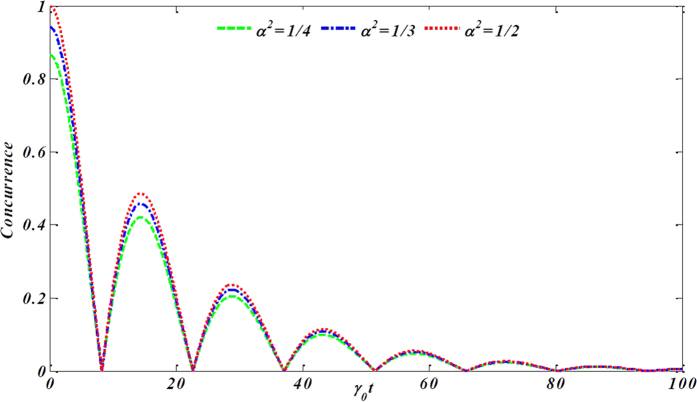
The dynamical behavior of concurrence versus the scaled time *γ*_0_*t* within the non-Markovian regime for the different initial states.

In Fig. 2, we plot the dynamics behaviors of concurrence *C*(*ρ*_*AB*_(*t*)) for the qubit subsystem as a function of the scaled time *γ*_0_*t* within the non-Markovian regime for the different state parameter *α*. It has been shown that the concurrence exhibits damped oscillations and suffers damped revivals at the critical time. At last, all of them gradually damp to a stable zero value.

### Mechanisms of entanglement revivals

In this section, we aim to explore the mechanism of the entanglement revival via an information-theoretic approach. The composite system illustrated in Fig. 1 can be described by a standard decoherence paradigm of a quantum system (qubit *A*) correlated with a measurement apparatus (qubit *B*) which in turn interacts with an environment (*E*)^37^,^38^,^39^. That is to say, the environment can also affect the qubit *A* via the qubit *B*. Then, we can divide the composite system 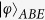 into three bipartite subsystems, namely, *AB, AE* and *BE*. To explore the intrinsic mechanisms, we will mainly focus on how the entanglement is distributed among all the bipartite constituents of 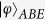 during the evolution. The corresponding flow of information presented in the composite system will be then investigated. Here we will employ the maximally entangled initial state.

**Figure 3:**
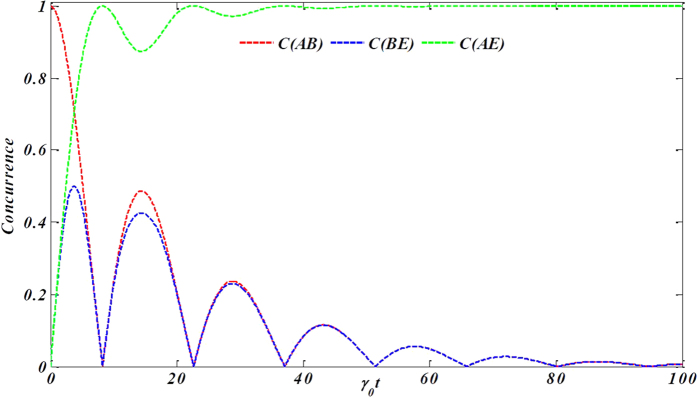
The redistribution of concurrence among bipartite systems contained in the hybrid system *φ*_*ABE*_ within the non-Markovian regime for the specific initial state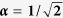.

Firstly, we show the distribution of the entanglement as Fig. 3. The two qubits are initially entangled and uncorrelated with the noisy environment in the beginning. From the Figure, one can readily see that the qubit subsystem-environment coupling reduces the initial entanglement *C*_*AB*_. It can be concluded that the entanglement flows from the subsystem {*AB*} to the subsystems {*BE*} and {*AE*}, in other words, both *C*_*BE*_ and *C*_*AE*_ arise immediately when the concurrence *C*_*AB*_ decreases. Moreover, *C*_*AB*_ and *C*_*AE*_ show a striking opposition behavior in the limit of finite time, such that the maxima of *C*_*AB*_ coincides with the minima of *C*_*AE*_, and vice versa. On the other hand, *C*_*AB*_ and *C*_*BE*_ behaviors are the similar each other in the most of the evolution. It deserves noting that the decrease of *C*_*AE*_ is not enough to compensate for the increase of *C*_*AB*_ or *C*_*BE*_ in the course of the first revival of *C*_*AB*_. Next, we will explicitly give our explanation for this. To do so, we will probe the flow of information, searching for a possible relation between bipartite entanglement and genuine tripartite correlations present in the composite system.

In this context, we use a recently introduced measure of genuine tripartite correlations^40^,^41^,^42^,^43^. Given a tripartite system {*A,B,E*}, the genuine tripartite correlations *υ*(*ρ*_*ABE*_) reads^44^


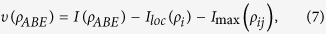


where


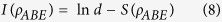


is the total state information measured by the mutual information living in the Hilbert space of dimension *d* with the von Neumann entropy *S*(•),


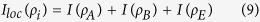


denotes the total information stored locally in each party with 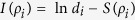, where *ρ*_*i*_ is the reduced density matrices of subsystem *i*, and


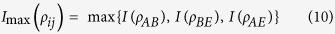


represents the maximal mutual information obtained over any possible bipartite reduced state, 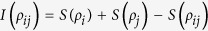. Physically, the Eq. (7) can be interpreted as follows: the total state information stored in a tripartite state is the sum of the information stored locally in each part, the maximal bipartite information in the system and its genuine tripartite correlations.

**Figure 4:**
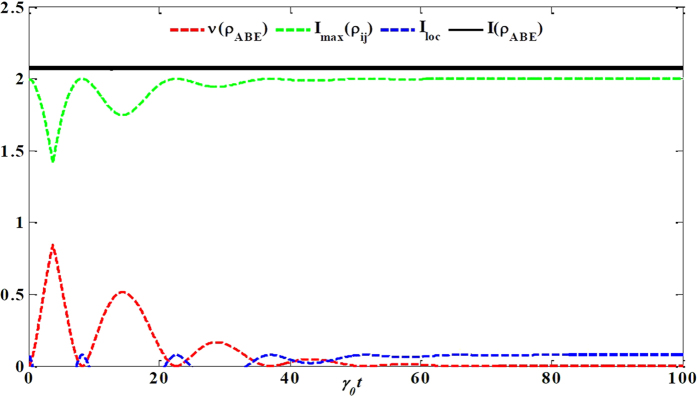
The information flow among the composite system *φ*_ABE_. Genuine tripartite correlations *υ*(*ρ*_*ABE*_), maximal bipartite correlations *I*_max_(*ρ*_*ij*_), local state information *I*_*loc*_ and total state information *I*(*ρ*_*ABE*_) versus the scaled time *γ*_0_*t* within the non-Markovian regime for the maximal initial state 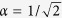.

Next, let us analyze the flow of information among all the constituents for our composite system. The dynamical evolution of all the relevant quantities involved in Eq. (7) versus *γ*_0_*t* within the non-Markovian regime is shown in Fig. 4. First of all, the total state information measured by *I*(*ρ*_*ABE*_) always keeps a constant value because the composite system is closed. Secondly, the information is stored in the bipartite, genuine tripartite correlations and/or local state information, which, in particular, is periodically transferred back and forth among them. Finally, more importantly, the maximal bipartite correlations *I*_max_(*ρ*_*ij*_) and the genuine tripartite correlations *υ*(*ρ*_*ABE*_) show a striking opposition behavior, i.e. the information stored in the *υ*(*ρ*_*ABE*_) transfers to the *I*_max_(*ρ*_*ij*_), which induces the revival of entanglement.

**Figure 5:**
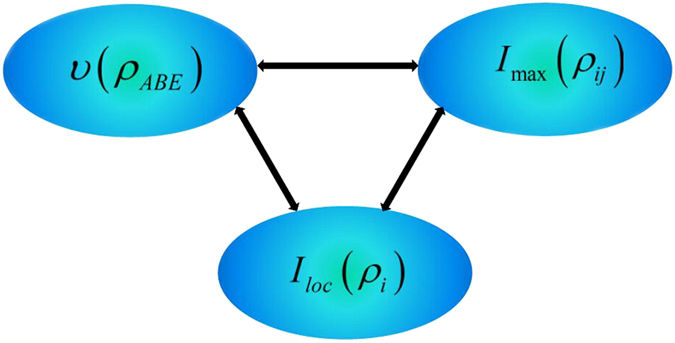
The sketch of the information flow among the genuine tripartite correlations *υ*(*ρ*_*ABE*_), maximal bipartite correlations *I*_max_(*ρ*_*ij*_) and local state information *I*_*loc*_.

To describe how the information flows among all constituents of the composite system we shall reanalyze its configuration. It turns out that *υ*(*ρ*_*ABE*_) is shared among three different subsystems: two qubit-subsystems and noisy environment. It is worth noting that *I*_max_(*ρ*_*ij*_) is a hybrid quantity since it may or may not involve the subsystem *E* during maximization of any possible two-party reduced states. Moreover, *I*_*loc*_(*ρ*_*i*_) is a manifest form of information stored in the system. On the other hand, *I*_*loc*_(*ρ*_*i*_) can affect dynamics of *I*_max_(*ρ*_*ij*_) and *υ*(*ρ*_*ABE*_) with certainty, due to the case that the local information stored in each party can evolve into bipartite and genuine tripartite correlations. At the same time, the bipartite correlations *I*(*ρ*_*AE*_), *I*(*ρ*_*AB*_) and *I*(*ρ*_*BE*_) can also affect the dynamics of *υ*(*ρ*_*ABE*_) or *I*_*loc*_(*ρ*_*i*_). To sum up, all of them can transform each other and the corresponding flow of information is shown in Fig. 5.

To attain a better quantitative understanding the information flow, we here will study the derivatives of three quantities 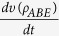, 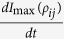 and 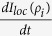. Particularly, during their evolutions there are two consecutive time-intervals, namely *T*^(1)^ = [0,t^*^] and *T*^(2)^ = [t^*^,*t*^*M*^], which are particularly imperative, with t^*^ being the time instant when the first maximum of *υ*(*ρ*_*ABE*_) is reached and *t*^*M*^ is the instant of the subsequent minimum of *υ*(*ρ*_*ABE*_). Having this in mind, one can obtain the explicit expressions of these derivatives in each time interval. Note that the two-qubit system and noisy environment are initially decoupled, such that the initial amount of correlations present in the overall system depends only on the qubit subsystem. So, in the interval *T*^(1)^ the maximal bipartite correlations is *I*(*ρ*_*AB*_) and 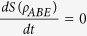 (total system is closed), then


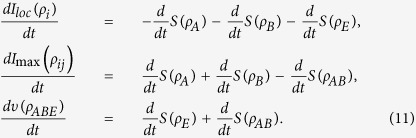


The above equations can quantify the information fluxes 
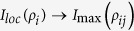
, 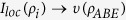 and 
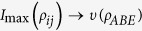
, which resort to the qualitative processes of Fig. 5. When one qubit of the quantum subsystem, viz., qubit *B* is coupled to the environment *E*, the two qubits leak information to the environment. Meanwhile, the leak can induce the environment to entangle itself with qubit *A*, which leads to the information flow into subsystem *AE*. Thus, we have 
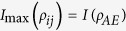
 in the second time interval *T*^(2)^, so that


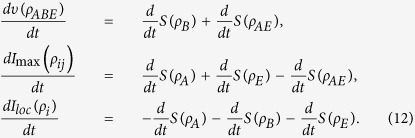


These equations can quantify the information fluxes 
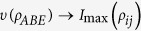
, 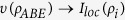 and 
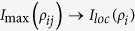
. After *t*^*M*^, due to the quasi-periodicity of the dynamics, all the information fluxes repeat new cycles.

## Conclusions

Here, we have investigated the dynamics behaviors of entanglement when the single qubit of bipartite subsystem interacts with a local non-Markovian environment. It turns out that the qubit subsystem-environment coupling can induce damped oscillations and revivals for the entanglement, which gradually decay to a stable value. Furthermore, by analyzing the correlation distribution and the flow of information among all the constituents of the composite system, we have illustrated the non-Markovian mechanisms of these revival behaviors. To gain a quantitative understanding of the information flow, explicit dynamical relationships among the genuine tripartite correlations, bipartite correlations and local state information via the methodologies of the corresponding derivatives are revealed. In particular, one or both of the genuine tripartite correlations and local state information periodically turn into the bipartite entanglement in the second time interval *T*^(2)^, which indeed induces the entanglement revival.

## Methods

### Proofs of the quantization for the local non-Markovian environment

From refs 19 and 30 we find that the non-Markovian environment can be actually quantized as the single-mode electromagnetic field in the high-*Q* cavity with an effective spectral density at zero temperature. Usually, the dynamics of the system-environment interactions can be described by master equations derived from perturbation theory.

To quantize the electromagnetic field in the high-*Q* cavity, it is convenient to begin with the classical description of the field based on the Maxwell’s equations. For simplicity, we do not list them here. The classical Hamiltonian for the electromagnetic field in a cavity resonator is^45^


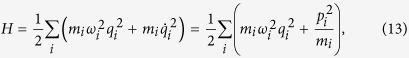


where *q*_*i*_(*i* = 1,2,3…) is the mode amplitude, 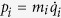 is the canonical momentum of the *i*th mode, *m*_*i*_ is a constant with the dimension of mass, and *ω*_*i*_ = *iπc*/*L* is the cavity eigenfrequency with *L* the cavity resonator length.

The present problem with respect to the quantization of the electromagnetic field in the high-*Q* cavity can be solved by identifying *q*_*i*_ and *p*_*i*_ as operators which obey the commutation relations


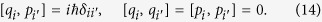


It is convenient to make a canonical transformation to the annihilation operator *a*_*i*_ and the creation operator 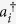



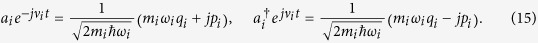


In terms of *a*_*i*_ and 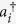, the Hamiltonian in Eq. (13) becomes


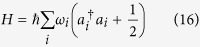


with the commutation relations between *a*_i_ and 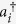



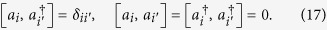


For the single-mode electromagnetic field, i.e., *i* = 1, the Hamiltonian is


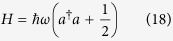


with the commutation relations [*a*,*a*^†^] = 1. Subsequently, by solving the Schrödinger equation one can obtain the eigenvalues and eigenstates of the Hamiltonian shown in Eq. (18). Therefore, it is turns out that one can achieve the quantization of the local non-Markovian environment.

A natural way to describe the dynamics of an open quantum system is to regard it as an interaction between the system and environment, which together form a closed quantum system. The dynamics of the closed quantum system can be described by a unitary transform. In general, based on the general notation 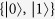 the dynamical map for the single qubit evolved in environment can be described as^1^


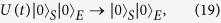



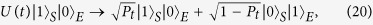


where *U*(*t*) is the time evolution operator of the environment, 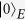 indicates the environment state where the mode is in a vacuum state, and 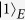 represents the environmental normalized collective state only containing one excited mode. The index *S* labels the quantum system. In many cases of practical interest it is reasonable to assume that the system and its environment are initialized in a product state. Now, assume that the system-environment input state is *ρ*_*S*_⊗*ρ*_*E*_, where the quantum system state is 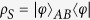 and the environment is in the vacuum state 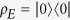. Then, the joint system evolves into


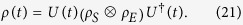


One can obtain the reduced density matrix for any bipartite subsystems by performing a partial trace over the remaining subsystem.

Moreover, the entanglement is quantified by the Wootters’ concurrence. For the *X*-type structures of the density matrix, we have a simpler expression^19^,^46^


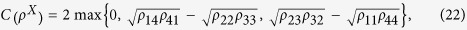


where *ρ*_*ij*_ are the elements of density matrix *ρ*^*X*^. Based on the definition in Eq. (22), one can derive the concurrence for different bipartite subsystems,


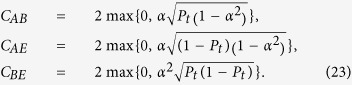


From the above equations, one can readily find that the bipartite concurrences *C*_*ij*_ are only the functions of state parameter *α* and environment strength *P*_*t*_. And *C*_*ij*_ can exhibit the distribution of entanglement among the different bipartite subsystems. Furthermore, we can also derive the genuine tripartite correlations *υ*(*ρ*_*ABE*_) for the tripartite composite system {*A,B,E*}, namely,


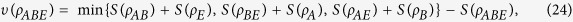


where *S*(*ρ*) = −Tr(*ρ* log_2_ *ρ*) is the von Neumann entropy. So we can get the desired derivatives of *υ*(*ρ*_*ABE*_) as shown in Eq. (11) and Eq. (12).

## Additional Information

**How to cite this article**: Shi, J.-d. *et al*. Entanglement revive and information flow within the decoherent environment. *Sci. Rep.* **6**, 30710; doi: 10.1038/srep30710 (2016).
