# Corrigendum: Entanglement revive and information flow within the decoherent environment

**DOI:** 10.1038/srep39371

**Published:** 2017-02-09

**Authors:** Jia-dong Shi, Dong Wang, Liu Ye

Scientific Reports 6: Article number: 3071010.1038/srep30710; published online: 08 10 2016; updated on: 02 09 2017

The authors neglected to cite a related paper that investigated quantum correlations and information flows in the presence of classical environments. This additional reference is listed below as reference 1, and should appear in the text as below.

In the **Results** section,

“To attain a better quantitative understanding the information flow, we here will study the derivatives of three quantities 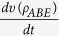, 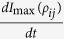 and 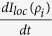.”

should read:

“To attain a better quantitative understanding the information flow, we here will study the derivatives of three quantities 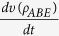, 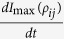 and 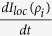 1.”
